# Inflammatory myofibroblastic tumor: a rare tumor of the lung

**DOI:** 10.3402/ecrj.v1.25390

**Published:** 2014-08-19

**Authors:** Özlem S. İçmeli, Levent A. Alpay, Baran Gündoğuş, Hatice Türker, Ayçim Şen

**Affiliations:** Süreyyapasa Chest Diseases and Thoracic Surgery Education and Research Hospital, Istanbul, Turkey

**Keywords:** lung, inflammatory tumor, myobibroblastic tumor, cancer, rare lung tumor

## Abstract

Inflammatory myofibroblastic tumor (IMT) is a rare lesion, representing 0.04–1.2% of all lung tumors. Brunn first described it in 1939, but its etiology remains uncertain. A 16-year-old patient was admitted to our hospital for further examination following abnormal radiological findings. The physical examination showed no abnormality, and routine hematological and biochemical parameters were within normal range. Chest radiograph revealed homogenous opacity of the right upper lobe with regular margins. Thoracic CT showed a nodular lesion, 30×26 mm in dimensions, with lobular contours in the right hilar. Bronchoscopic examination showed a vascular endobronchial lesion in the anterior right upper lobe, with bleeding when palpated. She underwent right thoracotomy for diagnostic and therapeutic purposes since bronchoscopic biopsy failed because of bleeding. With a pathological diagnosis of IMT, the present report discusses her case accompanied by relevant literature as it is a very rare type of lung tumor. IMT is a rare benign tumor. The diagnosis is difficult to make before surgery since its clinical and radiological features are variable and nonspecific. Although it is a benign lesion, it should be completely resected and patients should be closely monitored following the resection in order to avoid local invasion and recurrence.

Inflammatory myofibroblastic tumor (IMT) is a rare lesion, representing 0.04–1.2% of all lung tumors (1, 2). Brunn first described it in 1939, but its etiology remains uncertain (3, 4).

The diagnosis is difficult to make before surgical resection as clinical and radiological findings are nonspecific (2, 5).

## Case report

A 16-year-old patient who presented with a complaint of chronic cough was referred to our center for a lesion in her chest radiograph.

**Fig. 1:**
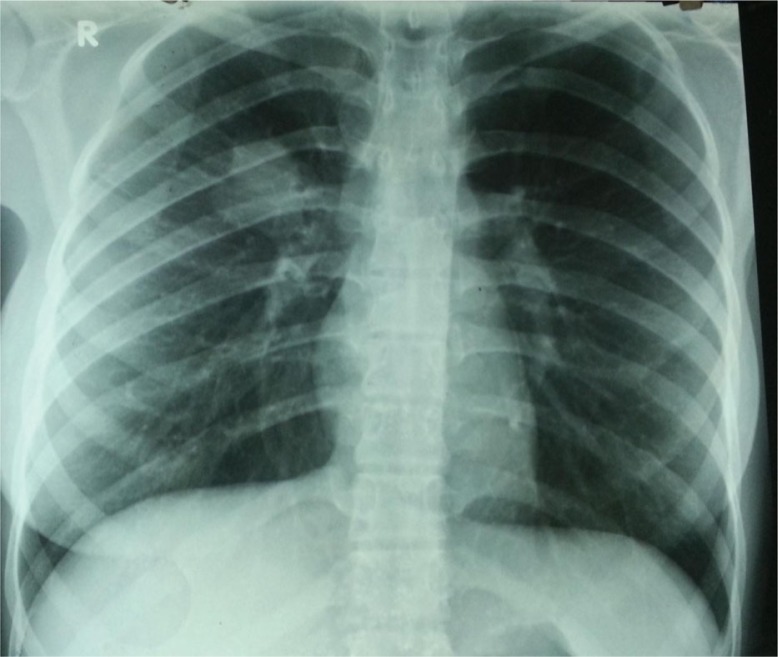
Initial chest radiograph.

**Fig. 2:**
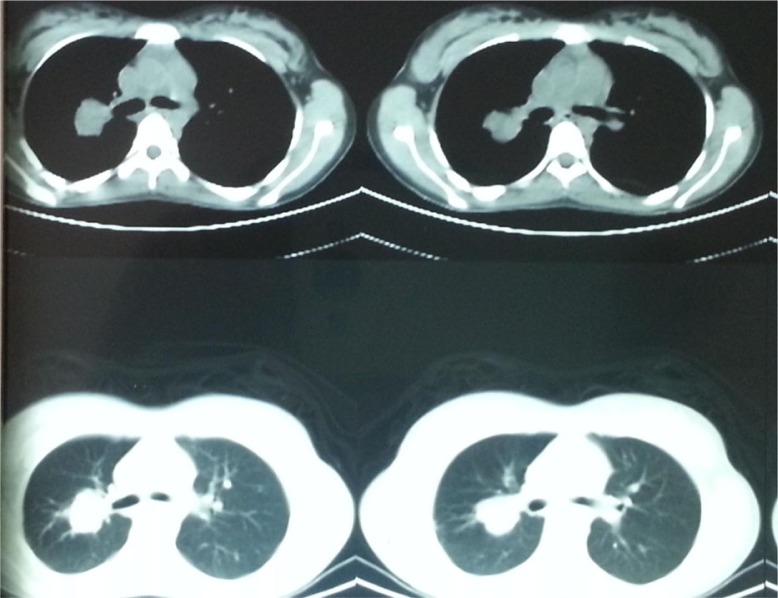
CT finding.

**Fig. 3:**
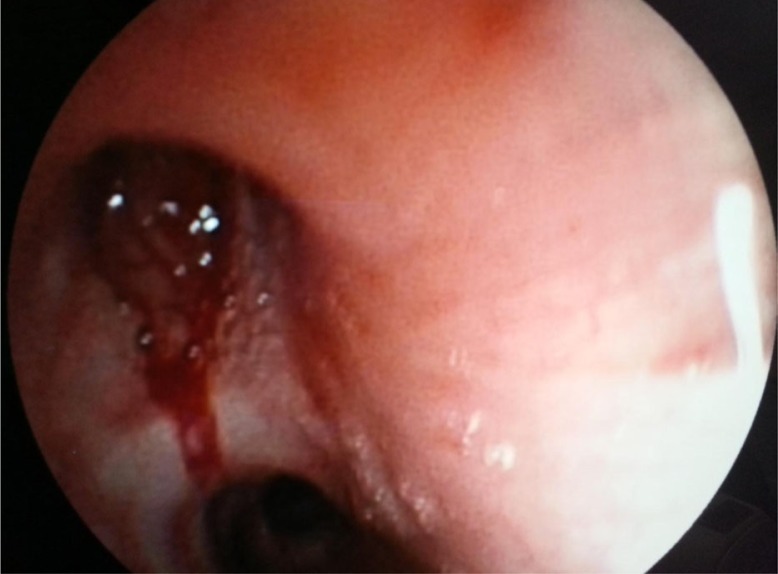
Bronchoscopic view.

**Fig. 4:**
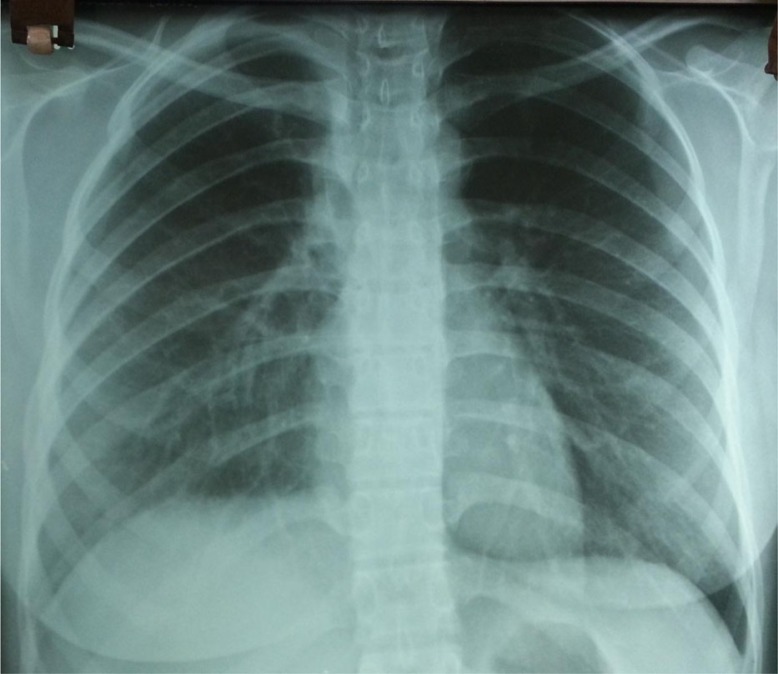
Postop radiograph.

The physical examination showed no abnormality, and routine hematological and biochemical parameters were within normal range. Chest radiograph revealed homogenous opacity of the right upper lobe with regular margins (Fig. 1). Thoracic CT showed a nodular lesion, 30×26 mm in dimensions, with lobular contours in the right hilar (Fig. 2). Bronchoscopic examination showed a vascular endobronchial lesion in the anterior right upper lobe, with bleeding when palpated (Fig. 3). She underwent right thoracotomy for diagnostic and therapeutic purposes since bronchoscopic biopsy failed because of bleeding. A right upper lobectomy was performed since the mass in the right upper lobe could only have been removed by lobectomy. A dirty white matter with a hemorrhagic center, measuring 4 cm in diameter, was observed macroscopically in the lobectomy material. Microscopic examination revealed formation of lymphoid aggregates with plenty of cells and plasma cells having an oval to fusiform and histiocytic appearance, which resulted in bundles and nodular clusters in patches. Immunohistochemical staining was negative for ALK and FL1-1, and positive for vimentin. The pathological diagnosis was reported as atypical IMT. The patient has no current complaint, and a postoperative chest radiograph showed no lesion (Fig. 4).

## Discussion

Representing 0.04–1.2% of all lung tumors, IMT is a rare lesion. It was classified by WHO in 1994 as a soft tissue tumor, which is also referred to as inflammatory pseudotumor, xanthoma, xanthomatosis, pseudotumor, plasma cell granuloma, fibrous histiocytoma or histiocytoma, and mast cell tumor or mast cell granuloma (2, 5, 6). It was first described in the lung in 1939 (3). While it usually occurs in the lungs, there are also reports of cases with extrapulmonary sites such as spleen, breast, maxillary sinus, epididymis, central nervous system, and soft tissues (6, 7). Lesions in central airways may lead to mechanical constriction, resulting in the development of postobstructive pneumonia and atelectasis. IMT affects both genders, and it is more common in children and young adults although it can occur at any age. In our case, tumor was detected in a very young age.

The pathogenesis of this lesion remains uncertain. Several hypotheses suggest that it may be associated with autoimmune or infectious mechanisms. It has been reported that in 30% of patients, IMT was related with recurrent infections caused by mycoplasma, nocardia, actinomycetes, and Epstein–Barr virus (5, 8). In our case, tumor was identified by morphological and immunohistochemical features, suggesting that it was likely to have been associated with recurrent infections.

Nonspecific symptoms such as cough, shortness of breath, hemoptysis, chest pain, fever, and fatigue may be observed in cases with IMT. However, such lesions may also be identified during routine screening or by a radiograph taken for other reasons in the absence of any symptoms (2, 7). Cough was the only symptom in our patient.

Radiological findings are variable and nonspecific. In 87% of patients, there is a mass or nodular lesion with regular margins, ranging from 1 to 6 cm in diameter. Nodules are usually solitary, but multiple nodules may develop occasionally. Our patient had a lesion with regular margins, measuring 4 cm in diameter in the upper right hemithorax. Calcification and cavitation are very uncommon. Ten percent of patients may have pleural effusion, while atelectasis may develop in 8% of patients. PET scan shows a positive FDG uptake like a malignant tumor (9). Our patient showed no evidence of calcification and cavitation, with a SUVmax of 6.7 in PET scan.

Complete resection is the treatment of choice for diagnostic and therapeutic purposes. The risk of recurrence is high in incomplete resections. The rate of local recurrence has been reported as 6.6–13% following the resection (10, 11). Corticosteroids are not recommended for use in adults, but they can be used in children with unresectable disease. Chemotherapy may be used for multifocal, invasive lesions and in cases of local recurrence. Following the complete resection, patients should be monitored at short intervals by bronchoscopy and thoracic tomography for any potential recurrence.

## Conclusion

IMT is a rare benign lesion. The diagnosis is difficult before surgery due to variable and nonspecific clinical and radiological features. The diagnosis is supported by histological and immunochemical examinations. Although it is a benign tumor, it should be completely resected, and the patients should be closely monitored in order to avoid any local invasion and recurrence.
